# Dietary patterns of patients with psoriasis at a public healthcare institution in Brazil^☆^^☆☆^

**DOI:** 10.1016/j.abd.2020.02.002

**Published:** 2020-05-12

**Authors:** Tatiana Cristina Figueira Polo, José Eduardo Corrente, Luciane Donida Bartoli Miot, Silvia Justina Papini, Hélio Amante Miot

**Affiliations:** a Department of Pathology, Faculdade de Medicina de Botucatu, Universidade Estadual Paulista, Botucatu, SP, Brazil; b Department of Biostatistics, Instituto de Biociências de Botucatu, Universidade Estadual Paulista, Botucatu, SP, Brazil; c Department of Dermatology and Radiotherapy, Faculdade de Medicina de Botucatu, Universidade Estadual Paulista, Botucatu, SP, Brazil; d Department of Nursing, Faculdade de Medicina de Botucatu, Universidade Estadual Paulista, Botucatu, SP, Brazil

**Keywords:** Feeding behavior, Food consumption, Psoriasis

## Abstract

**Background:**

Psoriasis is a chronic inflammatory disease with systemic repercussions and an association with comorbidities such as metabolic syndrome, cardiovascular diseases, and obesity. Psoriasis patients have a higher prevalence of obesity compared to the general population. Diet is a relevant environmental factor, since malnutrition, inadequate body weight, and metabolic diseases, in addition to the direct health risk, impair the treatment of psoriasis.

**Objectives:**

To evaluate food intake patterns, anthropometric, and metabolic syndrome-related aspects in psoriasis patients.

**Methods:**

Cross-sectional study through anthropometric assessment and food frequency questionnaire. Food frequency questionnaire items were evaluated by exploratory factor analysis and identified dietary patterns were analyzed by multivariate methods.

**Results:**

This study evaluated 94 patients, 57% female, with a mean age of 54.9 years; the prevalence of obesity was 48% and of metabolic syndrome, 50%. Factor analysis of the food frequency questionnaire identified two dietary patterns: Pattern 1 – predominance of processed foods; Pattern 2 – predominance of fresh foods. Multivariate analysis revealed that Patterns 1 and 2 showed inverse behaviors, and greater adherence to Pattern 2 was associated with females, eutrophic individuals, absence of lipid and blood pressure alterations, and lower waist-to-hip ratio and skin disease activity.

**Study limitations:**

Monocentric study conducted at a public institution, dependent on dietary memory.

**Conclusion:**

Two dietary patterns were identified in a Brazilian sample of psoriasis patients. The prevalence of obesity and metabolic syndrome were greater than in the adult Brazilian population. The fresh diet was associated with lower indicators of metabolic syndrome in this sample.

## Introduction

Psoriasis is a chronic, genetically based, and immunologically mediated inflammatory disease. It has systemic repercussions and is associated with comorbidities, such as metabolic syndrome (MS), arterial hypertension (AH), dyslipidemia, diabetes mellitus (DM), cardiovascular disease, malignant neoplasia, and affective disorders.^1^, ^2^

It affects about 1.31% of the Brazilian population and, despite not being contagious and having a benign course, it has an important economic impact, as well as an impact on the quality of life of patients.^3^

Psoriasis triggers an abnormal release of cytokines involved in the inflammatory process, including IFN-γ, IL-1, IL-6, IL-17, IL-8, and TNF-α, which promote a chronic systemic inflammatory state, favoring the development of comorbidities.^4^

There is a higher prevalence of obesity (34%) among patients with psoriasis when compared with the general population (18%); moreover, the higher the body mass index (BMI), the greater the risk of developing psoriasis.^5^ Likewise, there is an association between the increase in abdominal circumference (AC) and hip circumference (HC) and the onset of psoriasis, which corroborates its association with MS. MS comprises a set of risk factors for cardiovascular disease and is also related to systemic inflammation.^6^, ^7^

In addition to drug treatment, changes in eating behavior and physical activity emerge as potential strategies for adjuvant treatment of psoriasis in order to reduce comorbidities.^8^, ^9^ Diet is an environmental factor of high interest to patients, since malnutrition, inadequate body weight, and metabolic diseases, in addition to the direct risk they pose to the general health, impair the treatment of psoriasis.^10^, ^11^

Due to the lack of consensus and guidelines establishing a specific diet for these patients and considering that there are few studies that evaluated the food consumption of patients with psoriasis, it is necessary to characterize their nutritional profile and dietary patterns in order to outline dietary strategies aimed at improving the quality of the diet in parallel with the treatment of the disease.

This study had as its primary objective the assessment of food consumption patterns; secondly, it aimed to correlate food consumption with anthropometric and clinical data and with MS markers in patients with psoriasis treated at a public dermatology clinic.

## Methods

This was a cross-sectional study that included psoriasis patients seen at the Dermatology outpatient clinic of the Faculdade de Medicina de Botucatu – Universidade Estadual Paulista, Botucatu, SP (Brazil), from February to August 2019.

The diagnoses of psoriasis were established by a qualified dermatologist, and the study included adults (> 18 years) of both sexes with all clinical types and all stages of severity.

Sampling was carried out for convenience, recruiting consecutive, consenting patients during their medical consultations at the institution. Patients following specific diets (such as celiac), patients with malabsorptive syndromes or with limitations that would make dietary or anthropometric characterization impossible were not included.

All those who agreed to participate in the research signed an informed consent form. The project was approved by the institution's Research Ethics Committee (protocol No. 3,317,869).

### Study variables

The Food Frequency Questionnaire (FFQ), adapted to assess food consumption, was applied; patients were asked about the consumption of 76 types of food. This evaluation allowed the identification of the consumption of these foods over a period of one year, stratified as daily, weekly, monthly, and annual portions.^12^

Demographic and socioeconomic data, as well as duration of psoriasis treatment, were collected at the time of anthropometric and dietary assessment.

For anthropometric assessment, body weight and height were measured and later on calculation of the body mass index (BMI; kg/m^2^), the stratification for diagnosis was as folloows: malnutrition (<18.5), eutrophy (18.5–24.9), overweight (25–29.9), and obesity (≥30).^13^

Waist circumference (WC) was measured at the midpoint between the last rib and the iliac crest using a tape measure; HC was also measured to establish the waist-to-hip ratio (WHR = Waist [cm]/Hip [cm]), allowing the evaluation of parameters of android obesity and the risk for cardiovascular disease according to the classifications by sex and age.^14^

Food consumption (FFQ) and anthropometric assessments were performed by a nutritionist, after routine consultation at the dermatology outpatient clinic.

As all patients were under treatment, more detailed clinometric scores (such as the Psoriasis Area and Severity Index [PASI]) are impaired due to the treatment; therefore, disease activity was estimated by the presence/absence of skin and joint lesions at the consultation.^15^, ^16^

The routine clinical assessment and the clinical exams were used to assess the presence of MS using the criteria of the NCEP ATP III (National Cholesterol Education Program Expert Panel on Detection, Evaluation, and Treatment of High Blood Cholesterol in Adults) to evaluate the components of metabolic syndrome: presence of DM or fasting glucose ≥ 100 mg/dL; abdominal circumference > 102 cm for men and 88 cm for women; triglycerides ≥ 150 mg/dL; HDL < 40 mg/dL for men and <50 mg/dL for women; and blood pressure greater than or equal to 130/85 mmHg, or hypertension treatment.^17^

### Statistical analysis

Qualitative data were described as absolute and percentage values. Quantitative data were represented as means and standard deviations, or medians and quartiles (p25–p75) if normality was not observed in the Shapiro–Wilk test.^18^

Dietary data (FFQ) were tabulated and converted into daily food consumption, then subjected to an exploratory factor analysis to determine dietary patterns and individualized standardized adherence scores to each dietary pattern (ranging from −3 to +3). The analysis included those foods mentioned by at least 30% of the sample; in the factorial model, only those with a factor load > 0.3 were kept. Principal component analysis was used for the extraction of dietary patterns, evaluated by the KMO test (Kaiser–Meyer–Oklin), with varimax rotation.^19^

To explain the variations in the scores of adherence to each diet pattern, a generalized linear model (gamma distribution, log binding function) was adjusted using the anthropometric, demographic, and psoriasis variables (joint disease and cutaneous activity) as predictor variables. The *β* coefficient of the regression was used to estimate the association effect.^20^

Subsequently, the variables were analyzed in a multivariate manner based on the multiple correspondence analysis of two dimensions and were then arranged on the perceptual map. The dimension of the effect was estimated by the inertia in each dimension and the associations between the variables were represented by their geometric proximity.^21^ Continuous variables were categorized as tertiles.

The sample was calculated in order to represent variables present in up to 10% of a population of up to 300 patients with psoriasis, with a standard error of up to 5%.^22^ Two-tailed *p*-values ≤ 0.05 were considered significant. SAS for Windows, v.9.4 and IBM SPSS v. 25 were used for statistical analyses.

## Results

The study included 94 psoriasis patients; Table 1 presents the main clinical and demographic data. The low educational and family income, high prevalence of comorbidities (especially MS), obesity, and high risk for the onset of cardiovascular disease according to the WHR were noteworthy. All patients were under treatment, and the PASI ranged from 0 to 17.6; 84% presented skin lesions on the day of the interview.

**Table 1:** Demographic, socioeconomic, anthropometric, and clinical characteristics of patients with psoriasis interviewed at the dermatology clinic (*n* = 94).

Variables	Results
*Sex*^a^	
Female	54 (57)
Male	40 (43)

*Age (years)*^b^	54.9 (12.8)

*Educationalstatus*^a^	
Illiterate	3 (3)
Elementary school	51 (54)
High school	31 (33)
University education	9 (10)

*Family income (minimum wage)*^a^	
1	17 (18)
2	44 (47)
≥3	33 (35)

*Anthropometric profile*	
Weight (kg)^b^	84.8 (17.1)
Height (cm)^b^	1.7 (0.1)
BMI (kg/m^2^)^b^	31.1 (6.2)
Eutrophic (18.5–24.9)^a^	11 (12)
Overweight (25–29.9)^a^	38 (40)
Obesity (>30)^a^	45 (48)
Waist circumference (cm)^b^	109.0 (13.7)
Hip circumference (cm)^b^	104.2 (14.7)
WHR (cm)^b^	1.06 (0.15)

*Clinical data*	
Disease duration (years)^b^	15.7 (12.1)
Skin disease activity (plaques)^a^	75 (84)
Joint disease^a^	11 (12)
PASI^b^	5.3 (4.8)

*Comorbidities*^a^	
Triglycerides (mg/dL)	43 (49)
Cholesterol (mg/dL)	39 (43)
Hypertension (mmHg)	42 (47)
Altered blood glucose or DM (mg/dL)	34 (38)
Metabolic syndrome	47 (50)

DM, diabetes mellitus; WHR, waist-to-hip ratio; BMI, body mass index.

a *n* (%).

b Mean (SD).

As to systemic treatments, 32% used methotrexate, 23% used acitretin, and 45% were on immunobiological drugs (12% infliximab, 11% adalimumab, 10% secukinumab, 8% etanercept, 4% ustekinumab). There were no cyclosporine users in this sample.

Patients with psoriasis did not follow an exclusive diet, and all reported some variability in the food consumed. From the factorial analysis of the food consumption reported by the sample, two dietary patterns were identified (Table 2). Pattern 1 (processed diet) consisted of industrialized foods, rich in saturated fats, sugar, and sodium, *i.e.*, a diet with high caloric density and low nutritional quality. Pattern 2 (fresh diet) was characterized by the consumption of fruits and vegetables, which are sources of vitamins, minerals, fibers, and bioactive compounds, in addition to offering high nutritional support in qualitative terms.

**Table 2:** Food patterns and factorial loads of the foods that make up each pattern, identified by analyzing the sample food frequency questionnaire.

Pattern 1 (processed diet)	FL	Pattern 2 (fresh diet)	FL
Pizza	0.79	Raw vegetables	0.73
Deep fried savory snack	0.74	Cooked vegetables	0.73
Filled cookie	0.69	Tomato	0.70
Charcuterie	0.69	Cooked greens	0.68
Cheese	0.67	Broccoli	0.62
Sandwiches	0.66	Carrot	0.59
Soda	0.64	Lettuce	0.57
Flour	0.53	Orange	0.50
Burger	0.52	Salt	0.42
Mayonnaise	0.45	Apple	0.42
Baked savory snack	0.38	Olive oil/cooking oil	0.39
Breads	0.38	Banana	0.38
Desserts	0.37	Melon	0.38
Sausage	0.35	Papaya	0.38
Industrialized juice	0.32	Potato	0.31
Butter	0.32		

FL, factorial load; KMO = 0.65.

Adherence scores to each dietary pattern (Table 3) were assessed according to anthropometric, demographic, and clinical (joint and cutaneous activity) variables. Pattern 1 was associated with more recent psoriasis. The female sex showed greater adherence to Pattern 2, as well as patients with higher age and income. There was an inverse association of WHR with adherence to Pattern 2, as well as with cutaneous activity (plaques).

**Table 3:** Multivariate comparison of the scores of dietary patterns with anthropometric, demographic, and psoriasis-related variables (*n* = 94).

Variable	Pattern 1 (processed)		Pattern 2 (fresh)	
	*β* coefficient	*p*-Value^a^	*β* coefficient	*p*-Value^a^
*Female gender*	−0.027	0.748	0.268	0.006
*Age*	−0.01	0.123	0.05	0.045

*Educational status*				
College or university degree	0.042	0.760	−0.183	0.329
High school	−0.090	0.278	−0.069	0.547
Illiterate + elementary school	(−)			

*Income (minimum wages)*	0.020	0.327	0.099	0.003
*BMI*	−0.009	0.076	−0.009	0.492
*WHR*	0.230	0.362	−0.340	0.054
*Disease duration*	−0.005	0.034	−0.005	0.154
*Skin activity*	−0.110	0.233	−0.353	0.001
*Joint disease*	−0.145	0.096	−0.014	0.427
*Arterial hypertension*	−0.074	0.256	−0.037	0.852
*Diabetes mellitus*	0.092	0.208	0.176	0.142
*Hypertriglyceridemia*	−0.084	0.333	0.117	0.938
*Hypercholesterolemia*	0.187	0.057	−0.063	0.526

BMI, body mass index; WHR, waist-to-hip ratio.

a Adjusted *p*-value.

**Figure 1:**
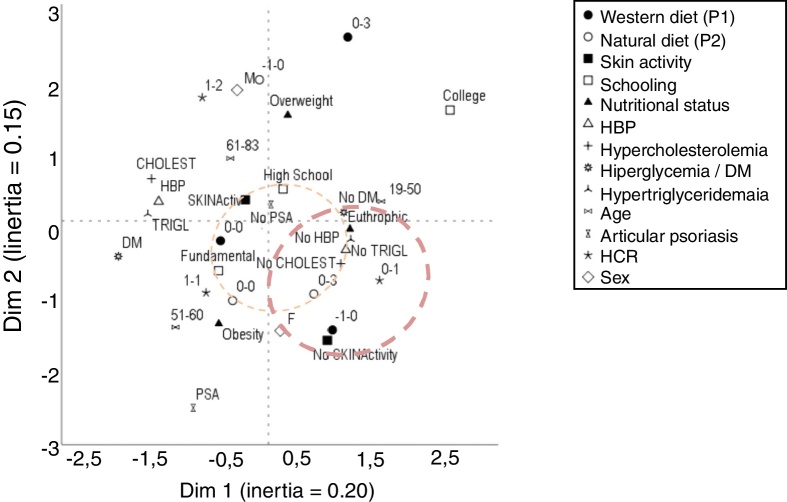
Perceptual map of the main demographic, clinical, and anthropometric variables and adherence to dietary patterns in 94 patients with psoriasis. Quantitative variables were categorized according to the tertile of the distributions. DM, diabetes mellitus; BMI, body mass index; WTH, waist-to-hip ratio; AH, arterial hypertension. Standardized scores for P1 and P2 ranged from −3 to +3 according to adherence to each dietary pattern.

The multivariate correspondence analysis (Fig. 1) indicated a relationship between lower adherence to the processed diet and greater adherence to the fresh diet, as well as with females, absence of lipid and blood pressure alterations, in addition to lower WHR and eutrophy. The presence of joint psoriasis was related with obesity.

## Discussion

Two patterns of food consumption were identified in patients with psoriasis, which were associated with clinical and anthropometric factors and MS markers.

Nutrition is a complex phenomenon, and since isolated foods or nutrients are generally not considered to be harmful to health, the assessment of the cumulative effect of diet on health through dietary patterns can help determine which are the most favorable for a population and provide a more accurate description of eating habits.^23^

Food patterns are transitioning worldwide, influenced by several factors, such as income, food costs, individual food preferences, beliefs, cultural traditions, and geographic, behavioral, and socioeconomic aspects.^24^

According to the Family Budget Survey (Pesquisa de Orçamento Familiar; POF 2018/2019), an increase in the consumption of foods with lower nutritional density, higher concentration of energy, total lipids, sugar, and sodium has been observed in Brazil over the past decades.^25^, ^26^ The most important changes in this regard were a reduction in the consumption of beans (31%), roots and tubers (32%), and eggs (84%), and an increase in the consumption of cookies (400%), soft drinks (400%), meats (50%), and milk (36%), *i.e.*, an increase in diets rich in processed and ultra-processed foods, characterizing the so-called Western diet, which is associated with increased obesity, MS, neoplasms, and inflammatory diseases.^27^, ^28^

The “Western diet” pattern has been a characteristic of the modern diet, with convenient, easily accessible, industrialized foods with low nutritional content.^8^ In the present study, adherence to the Western pattern (Pattern 1) was associated with overweight and presence of MS markers. All MS markers are modifiable risk factors; changes in lifestyle, diet, and exercise can favorably impact the onset of diseases.^25^, ^29^

In contrast, Pattern 2 was characterized by the consumption of fresh foods (fruits and vegetables), which are sources of vitamins, minerals, fibers, and bioactive compounds, in addition to offering a high nutritional intake. These are foods that provide micronutrient balance. Nutrients present mainly in fresh foods – such as fibers, vitamins, minerals, and mono- and polyunsaturated fatty acids – have antioxidant effects, which can reduce systemic inflammation and potentially interfere in the clinical picture of psoriasis and metabolic diseases.^9^, ^11^, ^27^

Cross-sectional studies have shown that a “healthy” food pattern is associated with a lower prevalence of MS, while western/unhealthy patterns are associated with an increased risk of MS.^30^ In this sample, the fresh pattern was associated with absence of alterations in the lipid profile and blood pressure, in addition to a lower WHR, evidencing that diets with a high fruit and vegetable content may mitigate the effects of inflammation and MS.^31^

A study conducted in Croatia with 82 inpatients found improvement in psoriasis plaques and reduction in total cholesterol, triglycerides, and low-density lipoprotein (LDL) after four weeks of adherence to a low energy density diet.^32^

An Italian study followed 61 patients for 24 weeks and observed weight and WC reduction, in addition to reduced PASI and serum levels of C-reactive protein, after a calorie-reduced diet combined with low-dose cyclosporine treatment.^33^

Another randomized clinical study, carried out in Denmark with 60 overweight psoriasis patients who adhered to a low-calorie diet for eight weeks, showed improvement in PASI and in the quality of life index when compared with the control group.^34^

In Sweden, a study with 51 patients evidenced the influence of the Mediterranean diet (rich in fish, olive oil, and vegetables); after three months of intervention, individuals showed improvement in disease activity and quality of life.^35^ These findings corroborate the results of a study carried out in Hawaii with five patients who showed improvement in PASI of 47.8% when submitted for ten days to a diet rich in fish, whole foods, fruits, vegetables, nuts, and herbal teas.^36^

In the present study, the prevalence of obesity was 48%. Obesity is a growing public health challenge, and is more prevalent among individuals with psoriasis (34%) than in the general population. In Brazil, over half of the population (55.7%) are overweight and 19.8% are obese (23% in women and 20% in men).^37^, ^38^, ^39^

Longitudinal population-based studies suggest a causal role of obesity in psoriasis, as well as an association with poor response to treatment and greater disease severity. The increase in obesity rates and adherence to the Western diet, in addition to longevity and greater access to diagnosis, may be associated with the increase in the incidence of psoriasis.^40^, ^41^, ^42^

Weight control can also improve the prognosis of psoriasis and quality of life. Moreover, caloric control leads to significant improvements in skin lesions and systemic inflammatory status. Therefore, low-calorie diets can be considered an important aid factor in the prevention and treatment of psoriasis, as well as in weight management, reduction of inflammatory markers, improvement in the lipid and glucidic profiles, and reduction of the risk of associated diseases.^43^, ^44^

A vegetarian diet, provided it is well planned and structured, can be nutritionally adequate and have potential health benefits, including for individuals with psoriasis.^32^, ^43^ A new line of research, on plant-based diets, prioritizes the consumption of foods of plant origin as natural and as close to their original form as possible, advocating the consumption of food in its most complete form, free from refinement, processing, and artificial additives. It seems possible that a plant-based diet is able to influence the immune and metabolic response from changes mainly in the intestinal microbial state.^45^ However, these interventions require studies with appropriate designs to clarify the relationship with psoriasis.^46^, ^47^

Nutritional strategies should be encouraged for patients with psoriasis; patients should be instructed to follow a low-calorie diet, with adjustment of macronutrients, micronutrients, and stimulated to eat fresh foods, restricting the consumption of processed foods and alcohol. Dietary interventions present positive results in the control of comorbidities and response to clinical treatment, and may help prevent diseases.^47^

In the present sample, the multivariate analyses reinforced the inverse correlation between MS markers and adherence to Pattern 1. Moreover, the association between diet, gender, income, and disease duration should be assessed in later studies with appropriate designs.

Study limitations include its monocentric and non-randomized design, including only a population of patients from a public institution, and the evaluation through the FFQ, which is memory-dependent.

The multidisciplinary care of patients with psoriasis allows a broader assistance aimed at reducing comorbidities along with the clinical treatment. The results of this study support the need for a specific nutritional care for psoriasis, aiming at possible improvement in disease activity and the reduction of risks arising from chronic inflammatory status and comorbidities.

## Conclusion

Two dietary patterns were identified in a sample of patients with psoriasis at a public healthcare institution. The prevalence of obesity and MS was significantly higher in this population than in the adult Brazilian population. Adherence to a fresh diet was associated with lower MS indicators.

## Financial support

None declared.

## Authors’ contributions

Tatiana Cristina Figueira Polo: Statistical analysis; approval of the final version of the manuscript; conception and planning of the study; elaboration and writing of the manuscript; obtaining, analyzing, and interpreting the data; effective participation in research orientation; intellectual participation in propaedeutic and/or therapeutic conduct of studied cases; critical review of the literature; critical review of the manuscript.

José Eduardo Corrente: Statistical analysis; obtaining, analyzing, and interpreting the data; critical review of the manuscript.

Luciane Donida Bartoli Miot: Approval of the final version of the manuscript; conception and planning of the study; elaboration and writing of the manuscript; obtaining, analyzing, and interpreting the data; intellectual participation in propaedeutic and/or therapeutic conduct of studied cases; critical review of the manuscript.

Silvia Justina Papini: Approval of the final version of the manuscript; conception and planning of the study; obtaining, analyzing, and interpreting the data; effective participation in research orientation; critical review of the manuscript.

Hélio Amante Miot: Statistical analysis; approval of the final version of the manuscript; conception and planning of the study; elaboration and writing of the manuscript; obtaining, analyzing, and interpreting the data; effective participation in research orientation; intellectual participation in propaedeutic and/or therapeutic conduct of studied cases; critical review of the literature; critical review of the manuscript.

## Conflicts of interest

None declared.
